# Dynamics of immune responses are inconsistent when trauma patients are grouped by injury severity score and clinical outcomes

**DOI:** 10.1038/s41598-023-27969-7

**Published:** 2023-01-25

**Authors:** Ya-Wen Yang, Che-Hsiung Wu, Huei-Ting Tsai, Ying-Ru Chen, Yu-Ping Chang, Yin-Yi Han, Tiffany E. Wu, Ray-Heng Hu

**Affiliations:** 1 Graduate Institute of Clinical Medicine, National Taiwan University College of Medicine, Taipei, Taiwan; 2 Department of Surgery, National Taiwan University Hospital, No.7, Chung Shan S. Rd. (Zhongshan S. Rd.), Zhongzheng Dist., Taipei, 100225 Taiwan; 3 Department of Traumatology, National Taiwan University Hospital, Taipei, Taiwan; 4 Division of Nephrology, Taipei Tzu Chi Hospital, Buddhist Tzu Chi Medical Foundation, Taipei, Taiwan

**Keywords:** Immunology, Physiology, Risk factors

## Abstract

The injury severity score (ISS) is used in daily practice to evaluate the severity of trauma patients; however, the score is not always consistent with the prognosis. After injury, systemic inflammatory response syndrome (SIRS) and compensatory anti-inflammatory response syndrome (CARS) are related to the prognosis of trauma patients. We aimed to evaluate the associations between the immune response and prognosis in trauma patients. Patients who admitted to the Trauma Intensive Care Unit (ICU) were eligible. Whole blood samples were collected at admission, and then 6, 12, 24, 48 and 72 h after admission. Natural killer (NK) cells, lymphocyte subset population and cytokines release were identified using flow cytometry. We grouped patients by their ISS (≤ 25 and > 25 as very severe injury) and ICU stay (≤ 10 days as a short ICU stay and > 10 days as a long ICU stay) for evaluation. Fifty-three patients were enrolled. ICU stay but not ISS was close correlated with activity daily living (ADL) at discharge. Patients with a long ICU stay had an immediate increase in NK cells followed by lymphopenia which persisted for 48 h. Immediate activation of CD8^+^ T cells and then exhaustion with a higher programmed cell death-1 (PD-1) expression and suppression of CD4^+^ T cells with a shift to an anti-inflammatory Th2 phenotype were also observed in the patients with a long ICU stay. When the patients were grouped by ISS, the dynamics of immune responses were inconsistent to those when the patients were grouped by ICU stay. Immune responses are associated with the prognosis of trauma patients, however the currently used clinical parameters may not accurately reflect immune responses. Further investigations are needed to identify accurate predictors of prognosis in trauma patients.

## Introduction

Trauma is a leading cause of mortality and morbidity worldwide, and it is also the leading cause of a loss of years of productive life. It is very heterogeneous in terms of the underlying causes, and it is characterized by considerable prognostic uncertainty. Although traditional vital signs such as blood pressure, heart rate, respiratory rate and body temperature are routinely monitored in trauma patients, their prognostic ability in these patients is unclear. The injury severity score (ISS) is widely used to evaluate trauma patients^1^. The ISS is calculated as the sum of the square of the three most severe injuries ranges from 3 (least) to 75 (most) injured. However, it only considers one injury per body region. An ISS of 1–8 is considered minor, 9–15 moderate, 16–24 severe, and 25 and higher very severe. ISS is widely used to predict mortality but is not linear^2,3^.

Following trauma, exaggerated innate immunity results in systemic inflammatory response syndrome (SIRS), and almost simultaneously, an appropriate and extremely important counter-inflammatory response is initiated known as compensatory anti-inflammatory response syndrome (CARS). The large amount of tissue injury caused by trauma releases various antigens and mediators. The response is sterile, and these endogenous factors interact with immune cells to initiate inflammatory responses^4^. SIRS commonly follows traumatic injury in humans and is the result of innate immune system activation^5,6^. The purpose of the inflammatory response is to limit further damage, clear debris and promote healing, and the degree of immune system activation is related to the concentration of cytokines produced^7^. CARS usually reflects all autoimmunosuppression caused by a major insult such as sepsis, burns, or tissue injury and is mediated primarily by the adaptive immune system and, in particular, by T cells. It is well-known that trauma alters the immune system, and that this process is complex and dynamic. However, the relationship between the dynamics of immunity and prognosis in trauma patients is uncertain.

Several studies^8–10^ have shown that short- and intermediate-term mortality outcomes were worse for patients who had prolonged stays in ICU. The definition of prolonged ICU stay is different by choosing different endpoint which results in the effect of a prolonged stay in ICU on prognosis in critically ill patients remains controversial and uncertain. One study defined prolong ICU stay as longer than 10 days because of the physiological insults of a critical illness occur within the first 10 days of the onset of a critical illness^11^.

Activity of daily living (ADL) is used as an indicator of a person’s functional status^12^ and the inability to perform ADLs results in the dependence of other individuals and/or mechanical devices. ADL is an important predictor of admission to nursing homes, need for alternative living arrangements, and use of paid home care which is related to the prognosis in trauma patients^13,14^. In general, the scores of 0–20 indicate “total” dependency, 21–60 indicate “severe” dependency, 61–90 indicate “moderate” dependency, and 91–99 indicates “slight” dependency^15^. Most studies apply the 60/61 cutting point.

The logistical challenges of conducting trauma research mean that few studies have specifically examined consecutive immune cell populations after injury, and few have focused on the first 2 h. Therefore, the aim of this prospective observational cohort study was to investigate associations between the immune response and prognosis in trauma patients.

## Methods

### Study design

This is a prospective study conducted from March 2018 to January 2021, patients older than 20 years admitted to the Trauma Intensive Care Unit (ICU) at the Department of Traumatology of National Taiwan University Hospital, the level I trauma center in Taipei, Taiwan, were screened. Those without systemic diseases (severe liver disease, known bleeding abnormality, etc.), substance abuse, concomitant use of drugs (anticoagulant medication, etc.), or smoking habit were enrolled. The exclusion criteria included: age < 20 years, transfer from another hospital, arrival > 120 min from injury, and those refused or couldn’t sign informed consent. Data including demographics, co-morbidities, trauma mechanism, severity of ISS, ICU stay, ICU and hospital mortality were collected. Activity of daily living (ADL) at discharge of survivors were also recorded for prognostic assessment. Laboratory data were measured daily as part of routine patient care. Blood samples for further evaluations were collected from ICU admission. Eight healthy volunteers (4 were males and 4 were females) were also recruited in the period for control group. This study was approved by the Institutional Ethical Committee of National Taiwan University Hospital (NTUH IRB No. 201702060 RINA). All procedures were performed in accordance with the Declaration of Helsinki. Informed consent was obtained in writing from each patient before inclusion in the study.

### Sampling and peripheral blood mononuclear cell (PBMC) isolation

Whole blood samples were collected into heparin–containing tubes from the trauma patients at admission, and then 6, 12, 24, 48 and 72 h after admission. PBMCs were isolated from the whole blood samples using the standard gradient centrifugation method.

### Flow cytometry

Cell subset analysis was carried out using flow cytometry with the following antibodies: FITC conjugated anti-CD8a, FITC conjugated anti-IL-4, PE conjugated anti-*IFN-γ*, PerCP/Cy5.5 conjugated anti-CD4, PE/Cy7 conjugated anti-CD3, PE/Cy7 conjugated anti-PD-1, APC conjugated anti-CD8a, APC/Cy7 conjugated anti-CD56 and APC/Cy7 conjugated anti-CD3. The staining protocol was performed following the instructions provided with each antibody kit. Fresh isolated PBMCs (1 × 10^6^ cells/ml supplemented with RPMI 1640) were incubated with phorbol-12-myristate 13-acetate (81 nM), ionomycin (1338.6 nM), and brefeldin A (5 μg/ml) for 3 h in a humidified incubator at 37 °C and 5% carbon dioxide to induce intracellular cytokine production. The cell surface/intracellular markers were stained and analyzed via flow cytometry. These markers could be used to separate T, CD4^+^ T, CD8^+^ T and natural killer (NK) cells. We used *IFN-γ* and IL-4 expression on CD4^+^ T cells to identify CD4^+^ Th1 and CD4^+^ Th2 T cells, respectively.

### Statistical analysis

Continuous variables were expressed as either the mean ± standard deviation (SD) or the median (interquartile range (IQR)), while categorical variables were presented as frequency and percentage. All clinical and flow cytometry data were analyzed using SPSS STATISTICS version 20 (IBM, Armonk, NY, USA). The Kruskal–Wallis H test and Mann–Whitney nonparametric U test were used for comparisons between groups. Categorical variables were examined using either the chi-square test or Fisher’s exact test. All tests were two-sided, and a 2-sided *p* value < 0.05 was considered to indicate a statistically significant difference.

### Ethics approval and consent to participate

This study was approved by the Institutional Ethical Committee of National Taiwan University Hospital (NTUH IRB No. 201702060 RINA). Informed consent was obtained in writing from each patient before inclusion in the study.

## Results

### Patients’ characteristics

**Figure 1:**
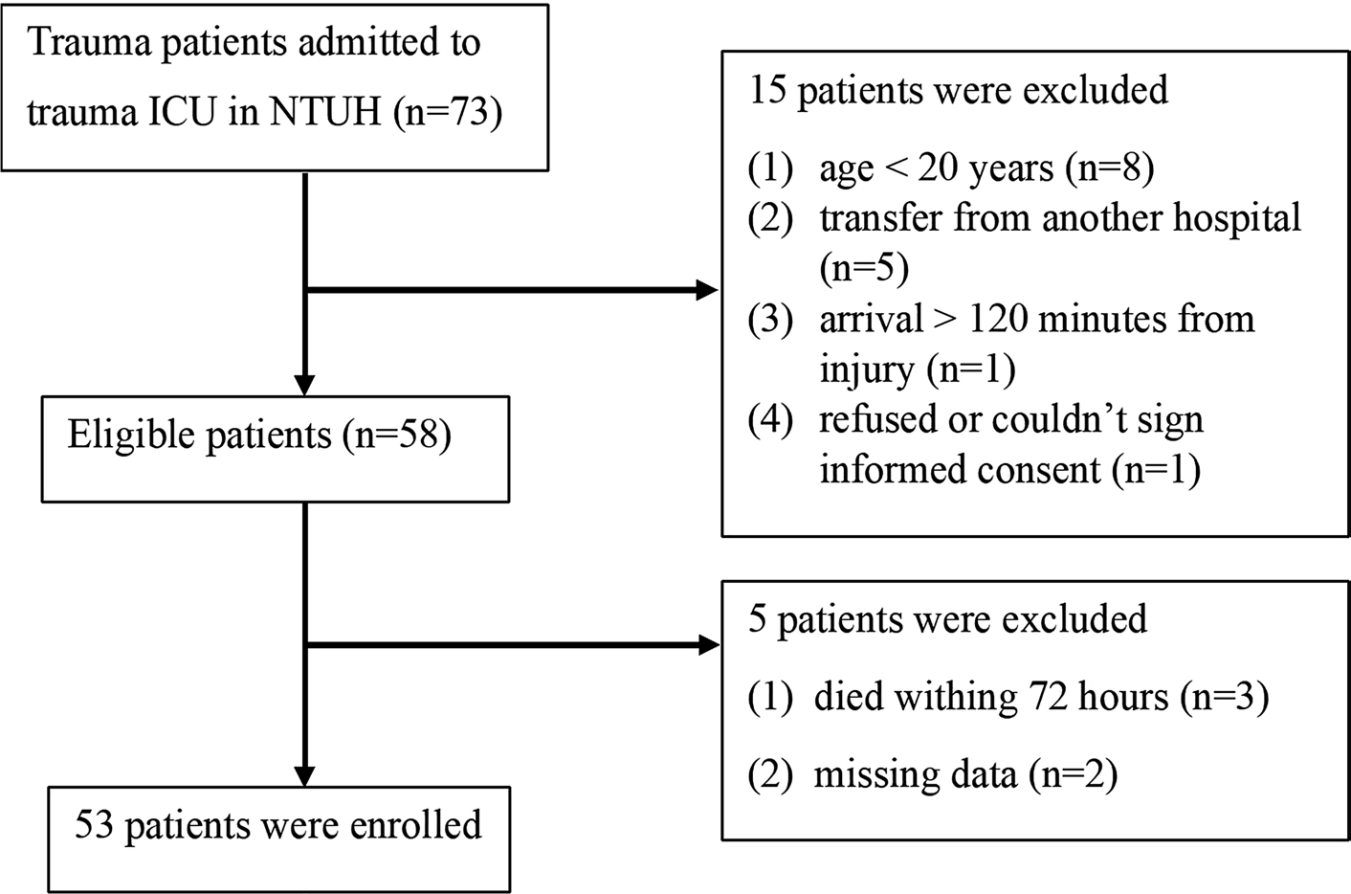
Patients’ recruitment chart.

Fifty-eight patients treated at our ICU were eligible for the study and five of them were excluded because of dying within 72 h and missing data (Fig. 1). Fifty-three polytrauma patients (mean age ± SD, 52.6 ± 19.5 years) were enrolled in the study. The ISS was 25 (IQR = 9). Thirty-seven patients (69.8%) were males. We first grouped patients according to their ISS. An ISS of 25 and higher is very severe injury, there were 31 patients had an ISS ≤ 25 and 22 had an ISS > 25. Five of the patients (9.4%) died during admission, one of whom had an ISS of 45 and the other four had an ISS of 25. The mortality rate was 12.9% in patients with an ISS ≤ 25 and 4.5% in patients with an ISS > 25. Among the 53 patients, 34 of them had traumatic brain injury. 96.2% of patients sustained blunt mechanism of trauma, with the top 2 leading causes of trauma were motor vehicle collision (69.8%) and falls (26.4%).

**Figure 2:**
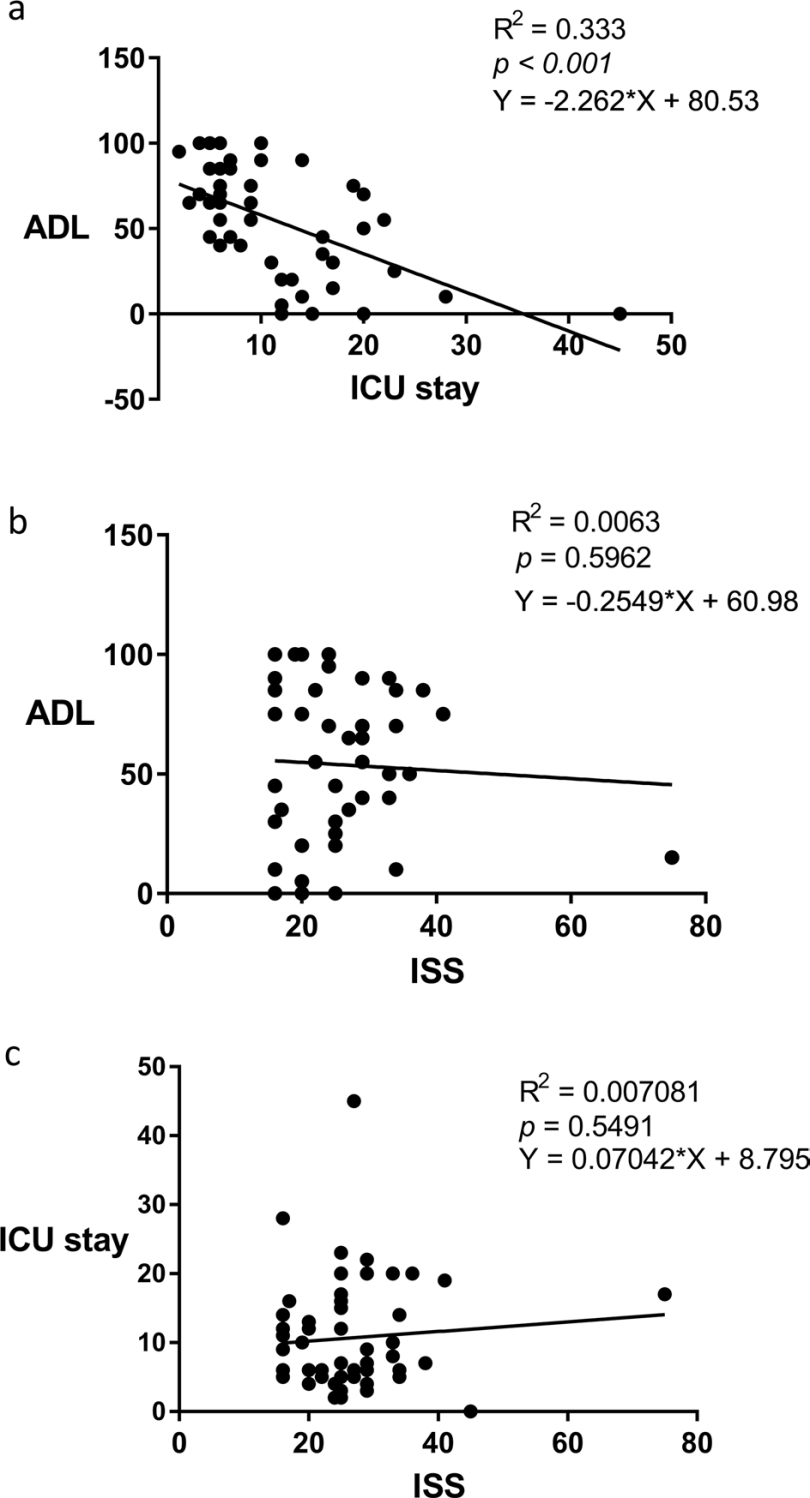
The correlation of ALD and ICU stay (**a**) and ISS (**b**) in trauma patients (survivors). (**c**) The correlation of ISS and ICU stay in trauma patients.

The ICU stay was 10.7 ± 8 days. There was strong correlation between ICU stay and ADL at discharge of survivors (Fig. 2a). On the other hand, there was no significant correlation between ISS and ADL at discharge (Fig. 2b). ICU stay was 9.1 days when we used the equation to find the best fit value of ADL 60 (Y =  − 2.262 × X + 80.53). We then grouped trauma patients as ICU stay longer than 10 days or not. Thirty-two patients had an ICU stay ≤ 10 days (short ICU stay) and the other 21 patients had an ICU > 10 days (long ICU stay). The patients who died were included in the long ICU stay group.

There were no significant differences in age, gender, type of injury and traumatic brain injury when the patients were grouped by either ISS or ICU stay. There was no significant difference in ICU stay when the patients were grouped by ISS, and there was no significant difference in ISS when the patients were grouped by ICU stay (Table 1). There was no significant correlation between ISS and ICU stay (Fig. 2c).

**Table 1:** Baseline characteristics of trauma patients grouped by ISS (A) and ICU stay (B).

(A)	ISS ≤ 25 (n = 31)	ISS > 25 (n = 22)	P value
ISS (median (IQR))	22 (9)	31 (5)	
Age (mean ± SD)	53.65 ± 20.07	51.06 ± 19.04	0.64
ICU stay (median (IQR))*	10 (10)	9 (13.5)	0.44
ADL (median (IQR))	45 (65)	65 (27)	0.052
Gender (male, %)	22 (71%)	15 (68.2%)	0.53
Types of injury			0.21
Motor vehicle collision	19	18	
Falls	11	3	
Others	1	1	
Traumatic brain injury (n, %)	21	13	0.36

(B)	ICU ≤ 10 days (n = 27)	ICU > 10 days (n = 26)	P value
ICU stay (median (IQR))	6 (2)	17 (6.5)*	
Age (mean ± SD)	47.98 ± 19.91	57.35 ± 18.24	0.08
ISS (medians/IQR)	25 (10)	25 (12.5)	0.38
ADL (median (IQR))	70 (35)	30 (40)	0.03
Gender (male, %)	16 (59.3%)	21 (80.8%)	0.14
Types of injury			0.6
Motor vehicle collision	21	16	
Falls	10	4	
Others	1	1	
Traumatic brain injury (n, %)	19	15	0.28

*Except the patients who died during admission.

### NK cells analysis

**Figure 3:**
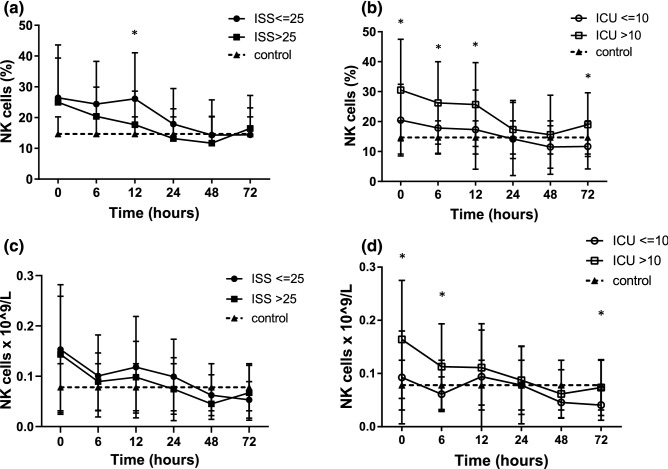
NK cells in trauma patients. (**a**) Patients with an ISS ≤ 25 had higher percentages of NK cells compared to those with an ISS > 25. (**b,d**) Patients with a long ICU stay had increased NK cells compared to that with a short ICU stay. (**c**) The NK cells in trauma patients grouped by ISS, *p < 0.05.

When the patients were grouped by ISS, those with an ISS ≤ 25 had significantly higher percentages (% of total lymphocytes) of CD3-CD56 + NK cells 12 h after ICU admission compared to those with an ISS > 25 (Fig. 3a,c). When the patients were grouped by ICU stay, the percentages and absolute numbers of NK cells were immediately increased after injury in the patients with a long ICU stay compared to those with a short ICU stay and the control group, and then returned to a normal level 24 h after injury (Fig. 3b,d).

### Lymphocyte’s analysis

**Figure 4:**
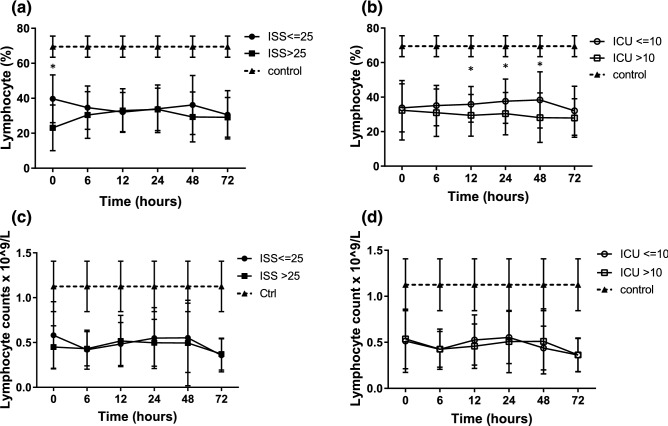
Trauma patients had lymphopenia compared to control ones. (**a,c**) Lymphocytes in trauma patients grouped by ISS. (**b**) The percentage of lymphocytes was significantly reduced in the patients with a long ICU stay compared to those with a short ICU stay. (**d**) The absolute numbers of lymphocytes between patients grouped ICU stay, *p < 0.05.

Trauma patients had lymphopenia when compared to the control group. When the patients were grouped by ISS, the percentage of lymphocytes (% of PBMCs) in the patients with ISS > 25 was lower compared to those with an ISS ≤ 25 at admission, but then became similar (Fig. 4a). However, when the patients were grouped by ICU stay, the percentage of lymphocytes in the patients with a long ICU stay was significantly lower from 12 to 48 h after ICU admission compared to those with a short ICU stay (Fig. 4b). There were no significant differences in the absolute numbers of lymphocytes between the patients grouped by ISS or ICU stay (Fig. 4c,d).

### T cells analysis

**Figure 5:**
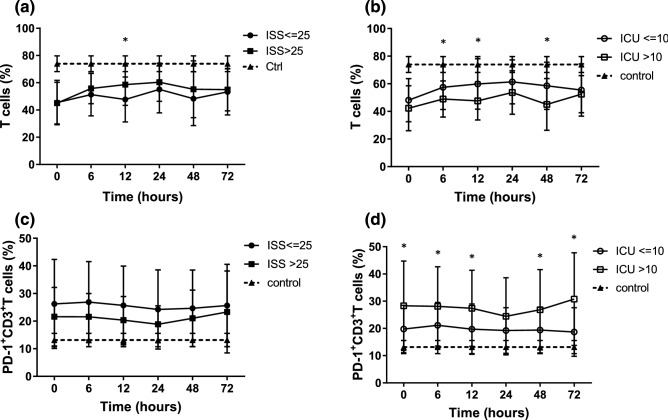
Trauma patients had suppressed T cells and increased expression of PD-1 on T cells compared to control group. (**a**) T cells in trauma patients grouped by ISS. (**b**) T cells were significantly suppressed in the patients with a long ICU stay compared to those with a short ICU stay*.* (**c**) PD-1 expression on T cells in trauma patients grouped by ISS. (**d**) PD-1 expression on T cells was significantly increased in the patients with a long ICU stay compared to those with a short ICU stay, *p < 0.05.

The trauma patients had a lower CD3^+^ T cells compared to the control group. When the patients were grouped by ISS, those with an ISS ≤ 25 had a lower percentage of T cells (% of lymphocytes) 12 h after ICU admission compared to those with an ISS > 25 (Fig. 5a). When the patients were grouped by ICU stay, those with a long ICU stay had a lower percentage of T cells (% of lymphocytes) from 6 to 48 h after ICU admission compared to those with a short ICU stay (Fig. 5b).

### Analysis of PD-1 expression on T cells

The expression of PD-1 on CD3^+^ T cells was higher in the trauma patients compared to the control group. When the patients were grouped by ISS, those with an ISS ≤ 25 had a higher PD-1 expression on CD3^+^ T cells compared to those with an ISS > 25, however there was no significant difference between the groups (Fig. 5c). When the patients were grouped by ICU stay, the PD-1 expression on CD3^+^ T cells was significantly higher in the patients with long ICU stay compared to those with a short ICU stay (Fig. 5d).

### CD4^+^ T cells analysis

**Figure 6:**
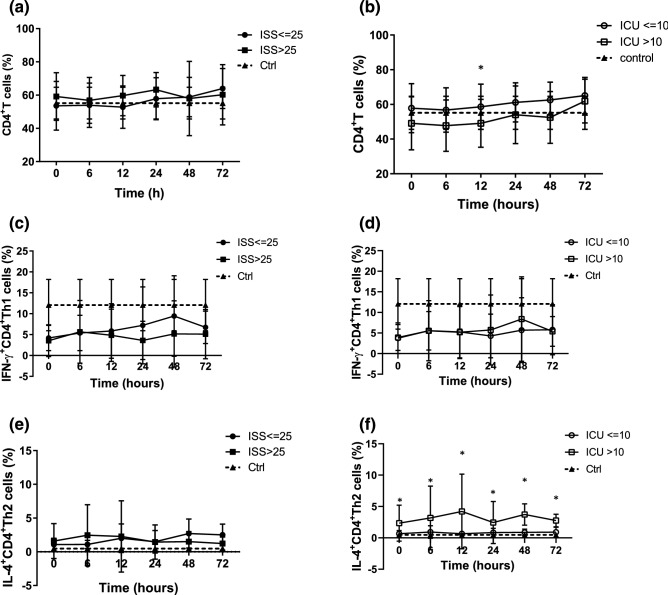
Trauma patients had CD4+ T suppression and shift to an anti-inflammatory Th2 phenotype. (**a**) CD4+ T cells in trauma patients grouped by ISS. (**b**) CD4+ T cells were more suppressed in the patient with a long ICU stay compared to those with a short ICU stay. (**c,d**) Trauma patients had suppressed IFN-γ production by CD4+ Th1 cells compared to the control group. (**e**) IL-4 production by CD4+ Th2 T cell in trauma patients grouped by ISS. (**f**) The patients with a long ICU stay had increased IL-4 production by CD4+ Th2 cells compared to those with a short ICU stay, *p < 0.05.

When the patients were grouped by ISS, those with an ISS ≤ 25 had reduced CD4^+^ T cells (% of T lymphocytes) compared to those with an ISS > 25, however there was no significant difference between the groups (Fig. 6a). When the patients were grouped by ICU stay, those with a long ICU stay had significantly reduced CD4^+^ T cells (% of T lymphocytes) compared to that with a short ICU stay at 12 h after ICU admission (Fig. 6b).

### Analysis of interferon gamma (IFN-γ) production by CD4^+^ Th1 cells

The trauma patients had lower *IFN-γ* production by CD4^+^ Th1 cells compared to the control group. When the patients were grouped by ISS or ICU stay, there were no significant differences in *IFN-γ* production by CD4^+^ Th1 cells between the groups (Fig. 6c,d).

### Analysis of IL-4 production by CD4^+^ Th2 cells

There was no significant difference in IL-4 production by CD4^+^ Th2 cells between the ISS groups (Fig. 6e). However, when the patients were grouped by ICU stay, those with a long ICU stay had significantly higher IL-4 production by CD4^+^ Th2 cells compared to those with a short ICU stay and the control group (Fig. 6f).

### CD8^+^ T cells analysis

**Figure 7:**
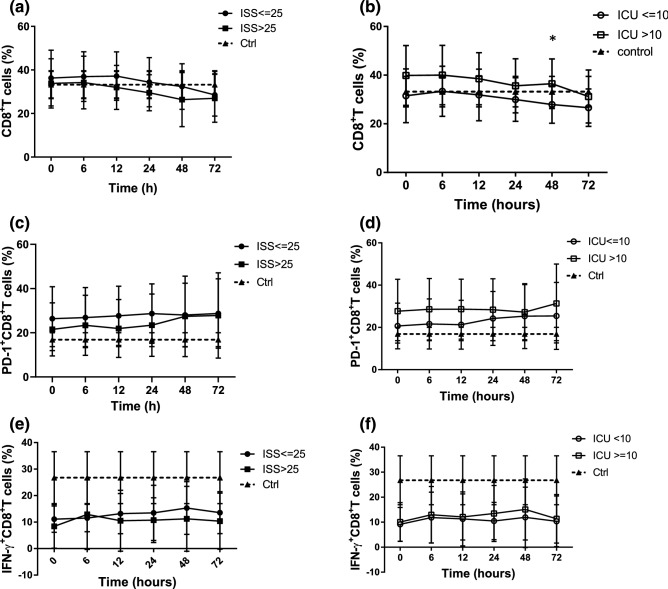
Trauma patients had increased PD1 expression on CD8+ T cells and reduced IFN-γ production by CD8+ T cells as compared to control group. (**a**) CD8+ T cells in trauma patients grouped by ISS. (**b**) The patients with a long ICU stay had an increased CD8+ T cells compared to those with a short ICU stay. (**c**) The expression of PD1 on CD8+ T cells in trauma patients grouped by ISS. (**d**) The patients with a long ICU stay had a higher expression of PD1 on CD8+ T cells compared to those with a short ICU stay. (**e,f**) IFN-γ production by CD8+ T cells in trauma patients grouped by ISS and ICU stay, *p < 0.05.

When the patients were grouped by ISS, those with an ISS ≤ 25 had an increased CD8^+^ T cells (% of T lymphocytes) compared to those with an ISS > 25 without significant differences (Fig. 7a). When the patients were grouped by ICU stay, those with a long ICU stay had a significantly increased CD8^+^ T cells (% of T lymphocytes) compared to those with a short ICU stay at 48 h after admission (Fig. 7b).

### Analysis of PD-1 expression on CD8^+^ T cells

The trauma patients had a higher expression of PD-1 on CD8^+^ T cells compared to the control group. When the patients were grouped by ISS, those with an ISS ≤ 25 had a higher expression of PD-1 on CD8^+^ T cells compared to those with an ISS > 25 (Fig. 7c). When the patients were grouped by ICU stay, those with a long ICU stay had a higher expression of PD1 on CD8^+^ T cells compared to those with a short ICU stay (Fig. 7d). There were no significant differences between groups when we grouped patients by ISS or ICU stay.

### Analysis of IFN-γ production by CD8^+^ cells

The trauma patients had reduced *IFN-γ* production by CD8^+^ T cells compared to the control group. When the patients were grouped by ISS or ICU stay, there were no significant differences in *IFN-γ* production by CD8^+^ T cells between the groups (Fig. 7e,f).

## Discussion

The ISS is used to evaluate the severity of trauma patients clinically. However, the mortality rate in patients with an ISS ≤ 25 was higher than that with ISS > 25 in our study. A prolonged ICU stay may be considered a risk factor for a poor prognosis and is close correlated with ADL at discharge. Trauma induces SIRS and CARS due to disorders of the innate and acquired immune responses. In this prospective study, we consecutively evaluated dynamics of immunity in trauma patients from ICU admission. Our findings showed that changes in immunity were inconsistent when the patients were grouped by ISS and ICU stay, and that dynamics of immunity could be useful predictors or potential therapeutic targets in trauma patients.

Trauma patients have great heterogeneity of etiology and severity. There are new clinical scoring systems developed for predicting the prognosis of trauma patients with certain kind of injury and severity^16,17^. Because of the patient group and severity in our hospital, there was no significant correlation between ISS and ICU stay. The dynamics of immunity is huge and important for the prognosis of trauma patients. Here we tried to find some parameters to provide us most information on these patients.

SIRS is the manifestation of immunoinflammatory activation that occurs in response to injury and released factors from disrupted tissue which induces cytokines, proinflammatory lipids, and related proteins. The presence of a systemic inflammatory response at admission has been associated with a poor outcome after trauma^18,19^. NK cells are lymphocytes in the same family as T and B cells and come from a common progenitor, however they play an important role in innate immune responses^20^. As cells of the innate immune system, NK cells are classified as group I innate lymphocytes and respond quickly to a wide variety of pathological challenges. Some studies have showed that number of NK cells increases immediately after trauma (2 h), possibly in response to neurotransmitters such as catecholamine^21^. This may reflect rapid mobilization from bone marrow and other secondary lymphoid tissues^22^. In our study, patients with an ISS ≤ 25 had significantly higher percentages of NK cells 12 h after ICU admission compared to those with an ISS > 25. However, the patients with a long ICU stay had a significantly higher NK cell expression compared to the patients with a short ICU stay from admission to 12 h and 72 h after admission. Cytokine concentrations have been shown to increase within 30 min of injury and can remain elevated for several days^23–25^. Persistence of this inflammatory response has been associated with multi-organ dysfunction syndrome (MODS) and a high possibility of death^26,27^. For trauma patients, rapid and persistent NK cell elevation may reflect exaggerated innate immunity leading to excessive SIRS and longer ICU stay.

**Figure 8:**
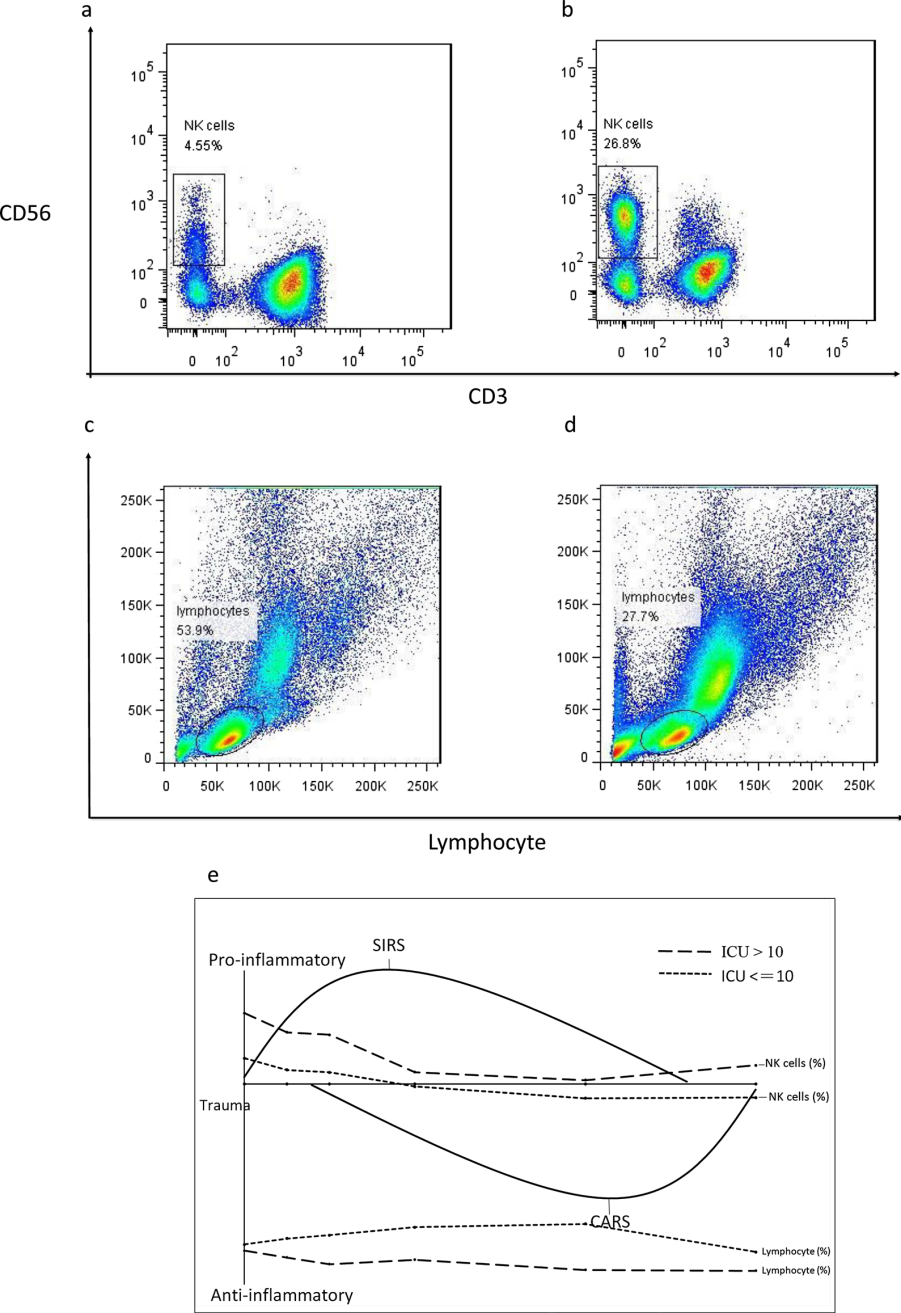
Trauma patients with long ICU stay had increased NK cells and lymphopenia compared to that with short ICU stay. (**a,c**) NK cells and lymphocytes in patient with short ICU stay. (**b,d**) NK cells and lymphocytes in patient with long ICU stay. (**e**) The SIRS, CARS and dynamics of immunity in trauma patients.

CARS is a systemic deactivation of the immune system tasked with restoring homeostasis from an inflammatory state. However, uncontrolled CARS can further result in immunoparalysis, impaired wound healing, recurrent nosocomial infections, and late MODS, all of which are associated with a poor prognosis. Lymphocytes can interact with innate and adaptive immune responses, and their energy or decreased responsiveness to stimuli has been demonstrated following major surgery, blunt trauma, and thermal injury. Lymphopenia itself is known to occur after severe injury, and a lack of lymphocyte recovery has been shown to impact survival^28^. Recent evidence suggests that lymphopenia develops within 24 h of injury, and that lymphopenia at 48 h is an early indicator of a poor outcome^29^. More of our trauma patients had lymphopenia than the healthy control, and significantly more of the patients with an ISS > 25 had lymphopenia compared to those with an ISS ≤ 25 on admission to the ICU, but the proportion of patients with lymphopenia became similar after that. When the patients were grouped by ICU stay, significantly more of those with a long ICU stay had lymphopenia compared to those with a short ICU stay from 12 to 48 h after admission. However, there were differences of percentages but not cell numbers of lymphocytes. It might be secondary impact of an expansion or decrease in a different cell type. Persistent lymphopenia reflected the critical condition of the patients and was related to a poor prognosis. SIRS and CARS are complex immunologic responses to injury. After injury, the patients with a long ICU stay had an immediate increase in NK cells followed by lymphopenia which persisted for 48 h (Fig. 8a–d). This indicates that rapid and persistent SIRS and subsequently severe CARS may be related to a prolonged ICU stay (Fig. 8e).

**Figure 9:**
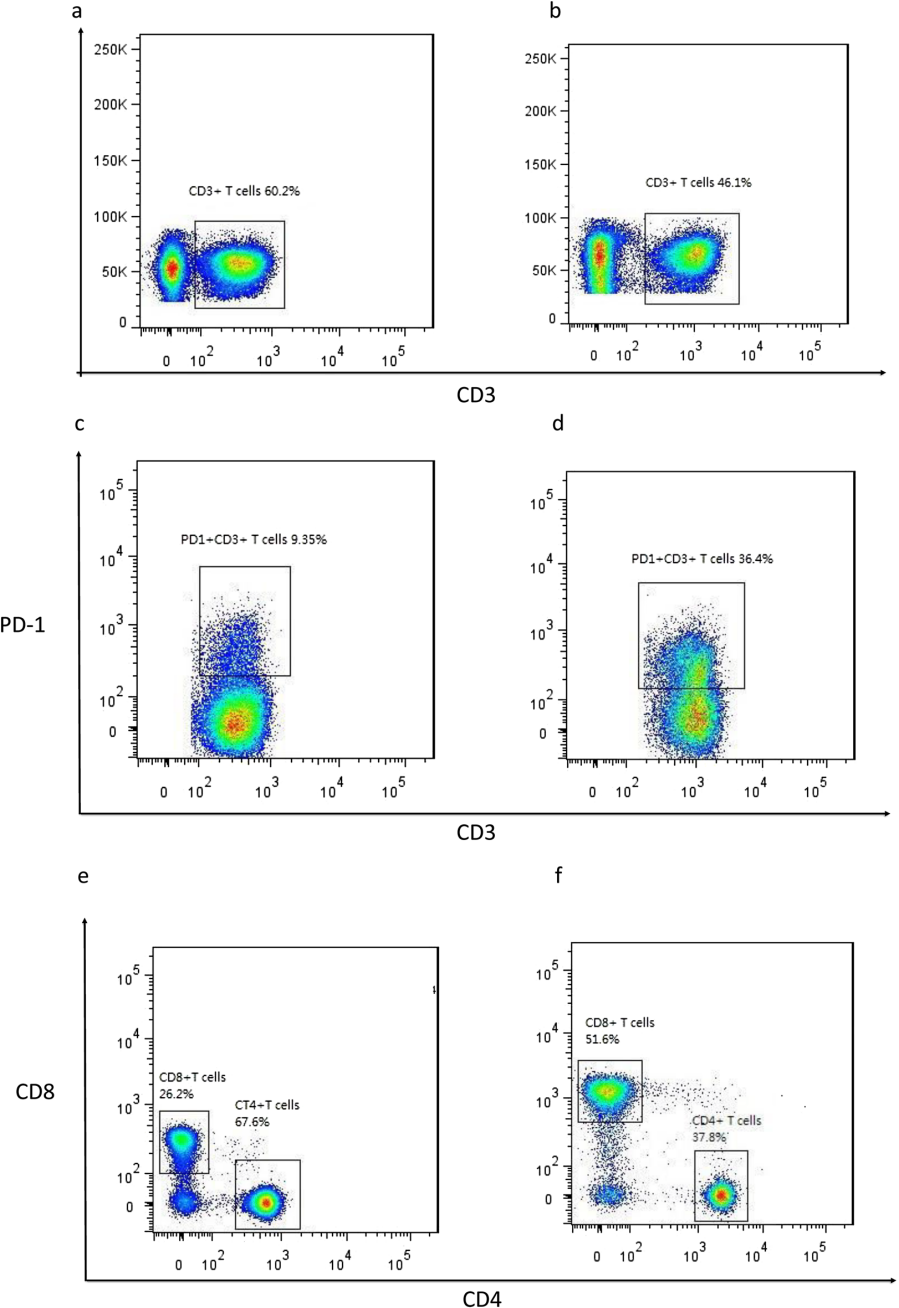
T cells and subpopulations in trauma patients. (**a,c,e**) T cells, PD-1 expression on T cells, CD4+ and CD8+ T cells in patients with short ICU stay. (**b,d,f**) T cells, PD-1 expression on T cells, CD4+ and CD8+ T cells in patients with long ICU stay.

As an essential component of adaptive immunity, T cells are involved in both cellular and humoral immunity. T-cell function is an important mechanism of immunosuppression observed after injury which may contribute to the development of complications^30^. The period of immunoparalysis after trauma is characterized by increased expressions of inhibitory coreceptors (PD-1, CD47, CTLA4) on T lymphocytes^31^. A high expression of PD-1 on T lymphocytes has been correlated with the severity of illness after major trauma^32^. In our study, the trauma patients had a lower T cells and higher PD-1 expression on T cells compared to the control group. When the patients were grouped by ISS, those with an ISS ≤ 25 had a greater reduction in T cells and increased PD-1 expression on T cells compared to those with an ISS > 25. When the patients were grouped by ICU stay, those with a long ICU stay had a significant reduction in percentages of T cells from 6 to 48 h after ICU admission and higher PD-1 expression on T cells compared to those with a short ICU stay (Fig. 9a–d). T cells may display limited function after injury as a result of exhaustion, which has been associated with the expression of some immune-inhibitory factors including PD-1 on T cell surface, and this has been related to a prolonged ICU stay and poor prognosis.

Injured cells release endogenous damage-associated molecular patterns into the circulation, which create a sepsis-like state^33^. Some studies have shown that trauma patients exhibit CD4^+^ T-cell loss with a relative increase in regulatory T-cell count, both of which are associated with unfavorable outcomes^34^. When the patients were grouped by ISS, a decrease in CD4^+^ T cells was observed in those with an ISS ≤ 25 compared to those with an ISS > 25 during the first 24 h after ICU admission without significant differences. When the patients were grouped by ICU stay, those with a long ICU stay had a significantly reduced CD4^+^ T cells compared to that with a short ICU stay at 12 h after ICU admission.

Beyond changes in numbers, circulating effector T lymphocytes also change from a pro-inflammatory Th1 phenotype to an anti-inflammatory Th2 phenotype after injury^35^. The impairment of effector helper T lymphocytes after trauma has also been shown to result in a reduction in *IFN-γ* production by Th1 polarized cells^36^. In our study, the trauma patients had suppressed *IFN-γ* production by Th1 cells compared to the control group. When the patients were grouped by ISS and ICU stay, there were no significant differences in *IFN-γ* production by CD4^+^ Th1 cells between the groups. IL-4 is produced primarily by mast cells, CD4^+^ Th2 cells, eosinophils and basophils, and further induces differentiation of naive helper T cells (Th0 cells) to Th2 cells. When the patients were grouped by ICU stay, those with a long ICU stay had significantly higher IL-4 production by CD4^+^ Th2 cells compared to the control group and those with a short ICU stay. For trauma patients, CD4^+^ T suppression and shift to an anti-inflammatory Th2 phenotype with increased IL-4 secretion reflect immunoparalysis and are associated with a prolonged ICU stay.

CD8^+^ T cells are important mediators of cytotoxic adaptive immunity. Some studies have reported no significant differences in CD8^+^ and CD4/CD8 ratio among healthy controls and patients with mild and severe trauma^37^. Another study reported that trauma plasma could induce an increase in CD8^+^ T cell and NK cell abundance, and the plasma from 1 day but not 3 days after trauma increased CD8^+^ T cells in PBMC cultures by manual gating^38^. When our patients were grouped by ICU stay, those with a long ICU stay had an increased CD8^+^ T cells compared to those with a short ICU stay at 48 h after admission. The T cell subpopulation analysis showed the patients with a long ICU stay had a reduced CD4^+^ T cells and increased CD8^+^ T cells 12 and 48 h after admission (Fig. 9e,f).

The inhibitory receptor, PD-1, plays a major role in CD8^+^ T cell exhaustion during chronic infections and cancer^39^, and is also expressed during the early phase of T cell activation when naive CD8^+^ T cells differentiate into effector cells^40^. In our study, the trauma patients had an increased expression of PD-1 on CD8^+^ T cells and reduced *IFN-γ* production by CD8^+^ T cells compared to the control group. The PD-1 expression on CD8^+^ T cells was higher in the patients with a long ICU stay compared to those with a short ICU stay. There were no significant differences in *IFN-γ* production by CD8^+^ T cells the patients were grouped by ISS or ICU stay. Immediate activation of CD8^+^ T cells after injury and then exhaustion was related to a prolonged ICU stay in the trauma patients.

There are several limitations of this study. First, because of the great heterogeneity in terms of etiology, mechanism, pathology, severity and treatment with widely varying outcomes, we could just enrolled polytrauma patients and most of them were suffered from blunt injury for evaluation. Second, because our hospital is a Level I trauma center, our patients had higher ISS than average. Third, some patients were excluded because they died before 72 h and didn’t have the data for all time points (Supplementary Table 1).

## Conclusions

Immune responses are closely correlated with prognosis. However, the dynamics of immune responses were inconsistent when the patients were grouped by ISS and ICU stay, indicating that the currently used clinical parameters may not accurately reflect immune responses. Understanding the dynamics of immunity could allow clinicians to stratify trauma patients and identify effective treatments in an acute setting to improve their management and prognosis^41^. Beyond the currently used scoring system, further evaluations should be conducted to identify an accurate predictor of prognosis in trauma patients.

## Supplementary Information

Supplementary Tables.

## Data Availability

The data sets used in this study are available from the corresponding author upon reasonable request.
