# Supplementary material for: Dynamics of immune responses are inconsistent when trauma patients are grouped by injury severity score and clinical outcomes
Source: Sci Rep. 2023 Jan 25;13:1391. doi: 10.1038/s41598-023-27969-7 (PMC9876923; doi:10.1038/s41598-023-27969-7)
Supplement: Supplementary file 1 — Supplementary Tables. [file 41598_2023_27969_MOESM1_ESM.pdf]

Supplements Table 1. Dynamics of immune responses in patients grouped by ISS.

	Control (n = 8)	ISS ≤ 25 (n = 31)	ISS > 25 (n = 22)	P value
**Percentage**				
NK cells (hour after admission)				
0 hour	14.69 ± 5.53	26.43 ± 17.16	24.99 ± 14.4	0.75
6 hours		24.38 ± 13.89	20.41 ± 9.53	0.26
12 hours		26.12 ± 14.97	17.68 ± 10.94	0.03
24 hours		17.91 ± 11.56	13.19 ± 9.65	0.12
48 hours		14.33 ± 11.49	11.68 ± 8.71	0.41
72 hours		14.38 ± 8.75	16.50 ± 10.75	0.49
Lymphocyte (hour after admission)				
0 hour	69.63 ± 6.08	39.68 ± 13.66	23.11 ± 13.05	<0.0001
6 hours		34.64 ± 12.39	30.47 ± 13.4	0.24
12 hours		32.09 ± 11.19	32.95 ± 14.25	0.79
24 hours		33.96 ± 13.61	33.63 ± 12.12	0.93
48 hours		36.17 ± 16.88	29.29 ± 14.25	0.15
72 hours		30.55 ± 13.83	29.09 ± 11.31	0.72
T cells (hour after admission)				
0 hour	73.97 ± 5.77	45.45 ± 16.36	45.07 ± 15.31	0.93
6 hours		51.32 ± 15.8	55.79 ± 11.14	0.27
12 hours		47.7 ± 16.52	58.60 ± 15.31	0.02
24 hours		55.09 ± 17.29	60.39 ± 14.02	0.25
48 hours		48.25 ± 19.79	55.18 ± 20.87	0.27
72 hours		53.26 ± 16.94	54.97 ± 15.56	0.74
PD-1 expression on T cells (hour after admission)				
0 hour	13.16 ± 2.42	26.23 ± 16.14	21.61 ± 10.59	0.24

6 hours		26.91 ± 14.7	21.56 ± 8.4	0.13
12 hours		25.67 ± 14.27	20.34 ± 8.57	0.13
24 hours		24.22 ± 14.33	18.83 ± 6.69	0.11
48 hours		24.66 ± 13.85	21.04 ± 10.23	0.34
72 hours		25.66 ± 14.93	23.33 ± 14.83	0.62
$CD4^+$ T cells (hour after admission)				
0 hour	55.18 ± 9.55	53.54 ± 14.71	59.14 ± 14.33	0.17
6 hours		53.88 ± 13.35	56.80 ± 13.83	0.45
12 hours		52.79 ± 12.78	59.67 ± 12.16	0.06
24 hours		57.81 ± 12.72	63.22 ± 10.34	0.11
48 hours		58.82 ± 11.89	57.96 ± 22.35	0.87
72 hours		63.95 ± 11.99	60.22 ± 18.12	0.43
$CD4^+$ Th1 cells (hour after admission)				
0 hour	12.06 ± 6.14	4.18 ± 3.02	3.54 ± 3.8	0.65
6 hours		5.39 ± 4.22	5.66 ± 7.51	0.92
12 hours		5.86 ± 6.52	4.83 ± 6.27	0.7
24 hours		7.21 ± 9.2	3.60 ± 4.6	0.22
48 hours		9.45 ± 9.63	5.16 ± 7.93	0.43
72 hours		6.73 ± 3.88	5.12 ± 5.91	0.64
$CD4^+$ Th2 cells (hour after admission)				
0 hour	0.48 ± 0.31	1.07 ± 0.94	1.60 ± 2.58	0.53
6 hours		1.10 ± 0.61	2.46 ± 4.53	0.36
12 hours		2.01 ± 2.13	2.27 ± 5.29	0.88
24 hours		1.49 ± 1.67	1.44 ± 2.54	0.96
48 hours		2.70 ± 2.15	1.51 ± 1.49	0.26
72 hours		2.51 ± 1.61	1.23 ± 0.98	0.14
$CD8^+$ T cells (hour after admission)				
0 hour	33.24 ± 6.26	36.28 ± 12.81	33.84 ± 11.32	0.48

6 hours		36.93 ± 11.41	34.22 ± 12.13	0.42
12 hours		37.18 ± 11.08	31.99 ± 10.12	0.09
24 hours		34.36 ± 11.33	29.51 ± 8.29	0.1
48 hours		32.37 ± 10.47	26.39 ± 12.4	0.09
72 hours		28.53 ± 9.7	26.99 ± 11	0.63
PD-1 expression on $CD8^+$ T cells (hour after admission)				
0 hour	16.88 ± 3.15	26.36 ± 14.46	21.52 ± 11.98	0.22
6 hours		26.90 ± 13.63	23.35 ± 13.64	0.37
12 hours		27.66 ± 13.41	21.93 ± 13.09	0.15
24 hours		28.67 ± 13.43	23.42 ± 14.11	0.19
48 hours		28.04 ± 14.42	27.41 ± 18.19	0.9
72 hours		28.71 ± 15.7	27.81 ± 19.33	0.88
IFN- γ production by CD8+ cells (hour after admission)				
0 hour	26.78 ± 9.78	11.12 ± 5	8.40 ± 8.27	0.34
6 hours		11.51 ± 5.16	12.91 ± 13.24	0.74
12 hours		13.19 ± 7.6	10.51 ± 11.53	0.511
24 hours		13.48 ± 10.43	10.71 ± 8.46	0.48
48 hours		15.32 ± 9.98	11.25 ± 12.27	0.55
72 hours		13.57 ± 7.9	10.37 ±10.59	0.61
**Cell count × 10^9/L**				
NK cells (hour after admission)				
0 hour	0.08 ± 0.05	0.15 ± 0.13	0.11 ± 0.1	0.23
6 hours		0.10 ± 0.08	0.08 ± 0.03	0.22
12 hours		0.12 ± 0.1	0.08 ± 0.05	0.14
24 hours		0.10 ± 0.07	0.06 ± 0.05	0.06
48 hours		0.06 ± 0.04	0.04 ± 0.03	0.06
72 hours		0.05 ± 0.4	0.06 ± 0.06	0.5

Lymphocyte (hour after admission)				
0 hour	1.13 ± 0.28	0.58 ± 0.38	0.45 ± 0.24	0.17
6 hours		0.42 ± 0.22	0.43 ± 0.19	0.86
12 hours		0.48 ± 0.24	0.52 ± 0.29	0.66
24 hours		0.55 ± 0.34	0.5 ± 0.26	0.57
48 hours		0.55 ± 0.39	0.49 ± 0.48	0.67
72 hours		0.58 ± 0.38	0.37 ± 0.18	0.81
T cells (hour after admission)				
0 hour	0.79 ± 0.16	0.30 ± 0.25	0.21 ± 0.17	0.19
6 hours		0.21 ± 0.13	0.25 ± 0.15	0.35
12 hours		0.25 ± 0.15	0.33 ± 0.24	0.2
24 hours		0.34 ± 0.25	0.32 ± 0.23	0.79
48 hours		0.28 ± 0.26	0.22 ± 0.19	0.39
72 hours		0.20 ± 0.13	0.21 ± 0.1	0.95

P values compare on patients with ISS ≤ 25 vs patients with ISS >25, using Student’s t test.

Supplements Table 2. Dynamics of immune responses in patients grouped by ICU stay.

	Control (n = 8)	ICU ≤ 10 days (n = 27)	ICU > 10 days (n = 26)	P value
**Percentage**				
NK cells (hour after admission)				
0 hour	14.69 ± 5.53	19.85 ± 12.48	31.17 ± 16.98	0.008
6 hours		17.75 ± 8.47	27.19 ± 13.63	0.005
12 hours		17.04 ± 13.26	26.65 ± 13.6	0.01
24 hours		14.12 ± 12.21	17.59 ± 9.66	0.25
48 hours		11.13 ± 6.97	15.79 ± 13.18	0.13
72 hours		11.08 ± 6.47	19.40 ± 10.41	0.003
Lymphocyte (hour after admission)				

0 hour	69.63 ± 6.08	33.78 ± 13.93	32.39 ± 17.21	0.74
6 hours		35.10 ± 11.65	30.93 ± 13.77	0.24
12 hours		35.85 ± 10.34	29.41 ± 13.06	0.04
24 hours		37.66 ± 12.8	30.41 ± 12.26	0.04
48 hours		38.35 ± 16.3	28.11 ± 14.38	0.03
72 hours		32.12 ± 14.24	27.93 ± 11.14	0.29
T cells (hour after admission)				
0 hour	73.97 ± 5.77	24.70 ± 12.1	41.63 ± 16.12	0.13
6 hours		26.10 ± 10.55	48.61 ± 12.81	0.03
12 hours		27.70 ± 12.17	46.91 ± 13.49	0.02
24 hours		27.24 ± 13.47	53.71 ± 15.75	0.13
48 hours		25.76 ± 11.5	45.05 ± 18.76	0.002
72 hours		20.53 ± 11.75	52.47 ± 13.44	0.57
PD-1 expression on T cells (hour after admission)				
0 hour	13.16 ± 2.42	20.05 ± 8.89	28.28 ± 16.97	0.03
6 hours		21.10 ± 8.39	28.10 ± 15.07	0.04
12 hours		19.50 ± 9.17	26.92 ± 13.99	0.03
24 hours		19.24 ± 8.4	24.42 ± 14.19	0.12
48 hours		19.38 ± 8.39	26.86 ± 14.75	0.04
72 hours		18.67 ± 8.92	30.75 ± 17.02	0.006
$CD4^+$ T cells (hour after admission)				
0 hour	55.18 ± 9.55	57.83 ± 14.1	52.13 ± 16.93	0.19
6 hours		56.78 ± 12.83	50.57 ± 16.02	0.13
12 hours		58.64 ± 13.07	50.84 ± 14.12	0.045
24 hours		61.19 ± 11.28	56.10 ± 16.79	0.21
48 hours		62.56 ± 10.27	55.64 ± 16	0.11
72 hours		65.12 ± 10.52	63.10 ± 12.2	0.57
$CD4^+$ Th1 cells				

(hour after admission)				
0 hour	12.06 ± 6.14	3.74 ± 3.74	3.90 ± 3.17	0.91
6 hours		5.55 ± 7.32	5.54 ± 4.68	0.1
12 hours		5.27 ± 6.49	5.20 ± 6.25	0.11
24 hours		4.30 ± 5.3	5.73 ± 8.51	0.62
48 hours		5.69 ± 7.87	8.39 ± 10.24	0.62
72 hours		5.79 ± 6.07	5.39 ± 3.6	0.91
$CD4^+$ Th2 cells (hour after admission)				
0 hour	0.48 ± 0.31	0.66 ± 0.51	2.34 ± 2.87	0.03
6 hours		0.92 ± 0.1	3.18 ± 5.07	0.12
12 hours		0.66 ± 0.29	4.22 ± 5.96	0.03
24 hours		0.83 ± 0.69	2.44 ± 3.35	0.09
48 hours		0.87 ± 0.51	3.72 ± 1.71	<0.0001
72 hours		0.91 ± 0.79	2.76 ± 1	0.007
$CD8^+$ T cells (hour after admission)				
0 hour	33.24 ± 6.26	31.54 ± 11.08	37.73 ± 13.26	0.07
6 hours		33.36 ± 10.31	38.17 ± 12.59	0.14
12 hours		31.89 ± 10.61	37.64 ± 10.61	0.06
24 hours		29.98 ± 8.94	34.30 ± 11.2	0.14
48 hours		27.95 ± 7.72	34.38 ± 10.97	0.03
72 hours		26.63 ± 7.72	30.38 ± 10.43	0.2
PD-1 expression on $CD8^+$ T cells (hour after admission)				
0 hour	16.88 ± 3.15	20.70 ± 10.81	27.68 ± 15.12	0.07
6 hours		21.60 ± 11.85	28.92 ± 14.39	0.06
12 hours		21.29 ± 11.56	28.64 ± 14.2	0.06
24 hours		24.23 ± 12.76	28.36 ± 14.59	0.3
48 hours		25.33 ± 15.36	28.01 ± 13.15	0.56

72 hours		25.43 ± 15.88	31.31 ± 18.67	0.31
IFN- γ production by CD8+ cells (hour after admission)				
0 hour	26.78 ± 9.78	8.23 ± 5.95	10.08 ± 7.77	0.31
6 hours		10.61 ± 9.32	12.97 ± 11.29	0.38
12 hours		10.48 ± 10.7	12.10 ± 9.2	0.53
24 hours		9.76 ± 7.24	13.50 ± 11.23	0.25
48 hours		9.60 ± 10.9	15.12 ± 12.22	0.25
72 hours		8.25 ± 8.9	11.34 ± 9.7	0.3
**Cell count × 10^9/L**				
NK cells (hour after admission)				
0 hour	0.08 ± 0.05	0.09 ± 0.09	0.16 ± 0.11	0.04
6 hours		0.06 ± 0.03	0.11 ± 0.08	0.01
12 hours		0.09 ± 0.1	0.11 ± 0.07	0.49
24 hours		0.08 ± 0.07	0.09 ± 0.06	0.65
48 hours		0.05 ± 0.03	0.06 ± 0.05	0.18
72 hours		0.04 ± 0.03	0.07 ± 0.05	0.02
Lymphocyte (hour after admission)				
0 hour	1.13 ± 0.28	0.52 ± 0.34	0.54 ± 0.32	0.82
6 hours		0.42 ± 0.19	0.43 ± 0.22	0.98
12 hours		0.53 ± 0.27	0.46 ± 0.24	0.37
24 hours		0.55 ± 0.28	0.51 ± 0.34	0.63
48 hours		0.44 ± 0.24	0.54 ± 0.41	0.34
72 hours		0.36 ± 0.18	0.36 ± 0.18	0.99
T cells (hour after admission)				
0 hour	0.79 ± 0.16	0.29 ± 0.23	0.25 ± 0.22	0.52
6 hours		0.24 ± 0.16	0.20 ± 0.12	0.28
12 hours		0.30 ± 0.23	0.22 ± 0.16	0.14

24 hours		0.36 ± 0.25	0.29 ± 0.24	0.33
48 hours		0.29 ± 0.2	0.24 ± 0.26	0.59
72 hours		0.20 ± 0.12	0.19 ± 0.12	0.78

P values compare on patients with ICU ≤ 10 days vs patients with ICU > 10 days, using Student's t test.
